# Adolescents’ Beliefs About Forced Sex in KwaZulu-Natal, South Africa

**DOI:** 10.1007/s10508-014-0280-8

**Published:** 2014-04-12

**Authors:** Hein De Vries, Sander Matthijs Eggers, Champak Jinabhai, Anna Meyer-Weitz, Reshma Sathiparsad, Myra Taylor

**Affiliations:** 1 Department of Health Education and Health Promotion, Maastricht University, POB 616, 6200 MD Maastricht, The Netherlands; 2 Department of Public Health Medicine, University of KwaZulu-Natal, Durban, South Africa; 3 School of Psychology, University of KwaZulu-Natal, Durban, South Africa; 4 School of Social Work and Community Development, University of KwaZulu-Natal, Durban, South Africa

**Keywords:** Gender, Sex differences, Forced sex, Rape, Self-efficacy, Social norms, South Africa

## Abstract

Gender-based violence has serious consequences for the psychological, physical, and sexual well-being of both men and women. Various gender roles, attitudes, and practices in South Africa create an environment that fosters submission and silence in females and hegemony and coercion in males. One of the expressions of this power inequity is a high prevalence of forced sex, which in its turn is associated with higher risk of HIV infection. This study therefore assessed potential gender differences in beliefs about forced sex and in prevalence of reported forced sex among high school students (*N* = 764) in KwaZulu-Natal. Results showed that significantly more boys were sexually active (26 %) than girls (12 %) and that boys experienced earlier sexual debut by over a year. Boys also held a more positive view about forced sex than girls since they associated it more often with signs of love, as an appropriate way to satisfy sexual urges, and as acceptable if the girl was financially dependent on the boy. The perception that peers and friends considered forced sex to be an effective way to punish a female partner was also more common among boys. On the other hand, boys were less knowledgeable about the health and legal consequences of forced sex, but no significant differences were found for other sociocognitive items, such as self-efficacy and behavioral intention items. Consequently, health education programs are needed to inform both boys and girls about the risks of forced sex, to convince boys and their friends about its inappropriateness and girls to empower themselves to avoid forced sex.

## Introduction

Gender violence includes verbal, emotional, physical, and sexual abuse, detracts from the health of both perpetrator and victim (WHO, 2002), and reduces the quality of life of the victims (Jewkes, Vundule, Maforah, & Jordaan, 2001). Rape, sexual coercion, and other forms of sexual violence are serious public health problems throughout the world. High rates of forced sex and other forms of sexual violence among women have been reported worldwide. Data suggest that, in some countries, nearly one in four women may experience sexual violence by an intimate partner and up to one-third of adolescent girls report their first sexual experience as being forced (Garcia-Moreno, Jansen, Ellsberg, Heise, & Watts, 2006; WHO, 2002, 2005).

Gender violence is prevalent in several countries (Garcia-Moreno et al., 2006). One of the countries with high prevalence rates is South Africa (Abrahams, Jewkes, Laubscher, & Hoffman, 2006; Dunkle et al., 2006; Pettifor, Hudgens, Levandowski, Rees, & Cohen, 2007). For South African adolescent girls, gender violence in intimate relationships plays an important role in their transition to first sexual intercourse (Harrison, Howerton, Secarea, & Nguyen, 2008; Jewkes, 2005; Jewkes et al., 2001; Reddy et al., 2003; Wood & Jewkes, 1998). Many children witness intimate partner violence in households where the female partner is at risk (Abrahams & Jewkes, 2005). A review by Jewkes and Morrell (2010) conclude that 25 and 55 % of South African women have experienced gender violence in their relationships.

Gender violence among South African adolescents has been documented extensively. Studies consistently show high rates of boys and girls reporting being forced into sex (Jewkes & Morrell, 2010; Mathews, 2008; Reddy et al., 2010; Sathiparsad, 2008). Jewkes and Abrahams (2002) reported in their review an incidence of 2,070 incidents per 100,000 women per year, with coerced forms of sex being a common problem in schools, workplaces, and among peers. Jewkes et al. (2006a) found that, among men aged 15–26 years, 16.3 % had raped a non-partner or participated in a form of gang rape and 8.4 % had been sexually violent towards an intimate partner. Jewkes and Morrell (2010) found that 42 % of men disclosed perpetration of intimate partner violence and 28 % disclosed rape of a woman or girl. Consequently, adolescent sexual health is regarded as among the most important health and development problems for South Africa (Dunkle et al., 2004; Jewkes & Abrahams, 2002).

Forced sex is related to several health consequences, such as teenage pregnancy risk and HIV infection (Jewkes & Morrell, 2010; Speizer et al., 2009). HIV-positive South-African women were more likely to report physical partner abuse than their sero-negative peers (Dunkle et al., 2004). In South Africa, the major route of HIV infection is through heterosexual transmission, with the epidemic affecting adolescents and young adults disproportionately. A South African national HIV survey in 2008 reported that, despite the decrease in HIV infections in other provinces, an 8 % increase in prevalence of HIV occurred among 15–24 year old adolescents in KwaZulu-Natal (Shisana et al., 2009). Moreover, the HIV/AIDS/TB epidemic has resulted in a reduction in lifespan of South Africans to an average age of 52 years (UNDP, 2010).

Several studies describe acceptance of gender violence in South Africa, not only by males but also by females (Sathiparsad, 2008; Wood & Jewkes, 1998). A study among Xhosa-speaking adolescent women revealed that male partners regularly used violence to define the conditions and timing of sex (Wood, Maforah, & Jewkes, 1998). The legitimacy of these coercive sexual experiences was reinforced by female peers who indicated that silence and submission was the appropriate response. Being beaten was such a common experience that some peers perceived this as an expression of love.

Research shows that forced sex beliefs and behavior are the result of several factors. Jewkes and Morrell (2010) conclude in a review about gender and sexuality that the norm of black Africans emphasizes toughness, strength, and expression of prodigious sexual success, and that it is such masculinity that women desire. Masculine men are expected to be in control of women and violence may be used to establish this control. Instead of resisting this, the dominant ideal of femininity embraces compliance and tolerance of violent and hurtful behavior, including infidelity. Besides masculinity, a wide array of other factors are also associated with forced sex, such as young age, alcohol use, drug use, previous experience with forced sex, number of sexual partners, gang membership, peer pressure to have sex, and income level (Flisher, Myer, Merais, Lombard, & Reddy, 2007; Jewkes & Morrell, 2010; Jewkes et al., 2006b; Morojele, Brook, & Kachieng’a, 2006; Pettifor, Measham, Rees, & Padian, 2004; Wubs et al., 2009).

One step in the approach to change forced sex beliefs and behavior requires the identification of important beliefs that are associated with forced sex. Although several South African studies have identified the beliefs associated with condom use (e.g., Dlamini et al., 2009; Godin et al., 2008; Mathews et al., 2009; Reddy, Meyer-Weitz, van den Borne, & Kok, 1999; Schaalma et al., 2009; Taylor, Dlamini, Nyawo, et al., 2007; Taylor, Dlamini, Sathiparsad, Jinabhai, & de Vries, 2007), very little information is available about beliefs and possible differences in beliefs about forced sex in boys and girls in KwaZulu-Natal. Yet, this information is essential to be able to guide future communication strategies aiming at changing perceptions and behavior concerning forced sex.

The first goal of this article was to describe prevalence rates and increases of prevalence rates with regard to forced sex among adolescents in KwaZulu-Natal. The second goal was to assess and compare beliefs about forced sex between boys and girls as well as to outline implications for future health communication strategies aimed at reducing favorable beliefs and behavior with regard to forced sex.

## Method

### Participants

This study used a sample of 772 adolescents taken from a randomized controlled trial (RCT) to reduce gender violence in 16 schools in KwaZulu-Natal (Taylor et al., 2011). Eight adolescents did not identify their gender at baseline, thereby reducing the sample to 764 students, of which 46.7 % were boys and 53.3 % were girls. The average age was 15.51 years (*SD* = 1.72) and almost a quarter did not live with their biological parents. The sample was relatively poor, given that 17 % of the students had experienced hunger before going to bed in the previous week.

At follow-up (after 8 months), 582 students (76 % of the original sample) completed our questionnaire, of which 386 were students that belonged to the control group of the RCT. Logistic regression analysis showed that being older (OR = 1.65, *p* < .001, 95 % CI 1.34–2.03) and being hungry before going to bed in the last week (OR = 1.86, *p* < .01, 95 % CI 1.22–2.83) were associated with drop-out within this group.

### Procedure

All schools were located in Ugu (population 790,000), a predominantly rural district. The government funded co-educational schools that participated in this study were randomly selected from lists obtained from the KZN Department of Education. Students completed a structured questionnaire at two time points: at the start of the study (T1) and 8 months thereafter (T2). For the purpose of this article, the identification of gender differences about beliefs and practices concerning forced sex, we will focus mainly on the cross-sectional results. Although the longitudinal data allowed us to depict the increase in forced sex and the distribution between boys and girls, there was insufficient power to conduct longitudinal regression analysis with the appropriate covariates. The completion of the questionnaire was facilitated by two teams of male/female pairs of young adults who introduced the study, explained the importance of valid answers, and emphasized the confidentiality of the students’ responses. The questionnaire took about an hour to complete in the classroom and on completion each student placed the questionnaire in a sealed envelope. No names were recorded and students did not receive compensation of any kind.

Ethical clearance to conduct the study was obtained from the University of KwaZulu-Natal’s Biomedical Research Ethics’ Committee and permission to undertake the study was obtained from the KwaZulu-Natal Department of Education and the principals at each school. Written informed consent was obtained from the parents and students. The questionnaires were coded to ensure confidentiality and students were provided with contact telephone numbers of organizations in their area assisting with sexual abuse.

### Measures

Focus group discussions were held with high school students to reveal their salient beliefs about forced sex. Based on these discussions and previous studies using the I-Change Model (De Vries et al., 2003; Dlamini et al., 2009), we identified relevant corresponding items. This resulted in an English questionnaire that was later translated into isiZulu (local language) for clarity and accuracy. Prior to being piloted to check understanding, we translated the questionnaire back into English to check for any inconsistencies due to the translation process.

*Sociodemographic information* assessed pertained to gender, age, ethnic background, religion, pocket money, and family status (see Table 1).

**Table 1:** Gender differences between boys and girls in demographics and sexual behavior

	Boys (*N*)	Girls (*N*)	Difference	*p*
Gender	46.7 % (353)	53.3 % (403)	–	–
Age (in years) (*M*, SD)	15.83 (1.76)	15.20 (1.61)	*t* (728) = 5.10	<.001
Religion				
Christian	41.2 % (136)	47.3 % (184)		
Traditional African	51.5 % (170)	45.5 % (177)		
Other	7.3 % (24)	7.2 % (28)	*χ* ^2^(2, 719) = 2.83	ns
Living with				
Mother and father	38.4 % (134)	33.3 % (133)		
Mother	38.1 % (133)	36.3 % (145)		
Father	3.4 % (12)	3.8 % (15)		
Other	20.1 % (70)	26.6 % (106)	*χ* ^2^(3, 748) = 4.89	ns
Pocket money				
No pocket money	32.0 % (103)	36.4 % (131)		
R1–R10	17.1 % (55)	11.9 % (43)		
R11–R20	5.3 % (17)	5.3 % (19)		
R21–R39	16.5 % (53)	17.2 % (62)		
R40 and over	29.2 % (94)	29.2 % (105)	*χ* ^2^(4, 682) = 4.14	ns
Number of times went to bed hungry in the last week				
None	77.5 % (220)	89.0 % (315)		
Once	8.1 % (23)	3.7 % (13)		
More then once	14.4 % (41)	7.3 % (26)	*χ* ^2^(2, 638) = 15.51	<.001
Sexually active	25.9 % (90)	12.0 % (48)	*χ* ^2^(1, 749) = 23.92	<.001
Age at sexual debut (*M*, SD)	14.03 (3.06)	15.34 (2.87)	*t*(129) = 2.36	.02
Pregnancy/made pregnant	2.0 % (7)	6.3 % (25)	*χ* ^2^(1, 744) = 7.98	<.001
Forced sex attempt	5.3 % (18)	7.2 % (28)	*χ* ^2^(1, 725) = 1.06	ns
Forced sex	4.9 % (17)	3.3 % (13)	*χ* ^2^(1, 741) = 1.21	ns

*Sexual experience* was measured by one question: Have you ever had sex? (yes/no). Forced sex was assessed using two questions: First, students were asked: Has anyone ever had sex with you against your will? (yes/no). A second question was: Did anyone have sex with you against your will in the last 6 months? (yes/no). If a student answered affirmative on either of these two questions, he or she was coded as being forced to have sex. With this cross-validation check, we were able to fill in some missing values.

*Knowledge* about forced sex and subsequent legal procedures was assessed with four statements: Forced sex is rape and is illegal in South Africa; There are special procedures to follow if your report that you have been raped; If you are raped, you should report what happened to the police who must open a case; and if you are forced to have sex, you may be at risk of HIV. Participants could answer with yes, no or I don’t know (Cronbach *α* = .76). The responses were then recoded into the proportion of correct answers given, ranging from 0 (no correct answers) to 1 (all answers correct). ‘I don’t know’ was always recoded as incorrect. On average, participants answered 77 % of the questions correctly.

*Beliefs about forced sex* assessed nine advantages (*α* = .87) and four disadvantages associated with forced sex (see Table 2 for a summary of the items; *α* = .93), 14 questions about perceived social influence about forced sex (*α* = .92), six questions about self-efficacy beliefs about not to engage in forced sex (*α* = .96), six questions about intentions not to engage in forced sex (*α* = .92), one cue to action, and two questions about future action planning (for a summary of the items, see Tables 3 and 4). Beliefs, social influence, self-efficacy, intention, cue to action, and action planning were all scored on a 5-point Likert scale, ranging from strongly disagree (1) to strongly agree (5). We did not calculate scales from these items, since we were interested in the specific topic that each single item covered. Single items were therefore used in the analyses.

**Table 2:** Gender differences in attitudinal beliefs concerning forced sex

	Boys		Girls		*F*	*p*
	*M*	SD	*M*	SD		
Attitude: beliefs that forced sex…						
Lets boys show that they really love their partner	2.51	1.43	1.99	1.17	*F*(1, 291) = 7.91	.005
Helps boys to satisfy their sexual urge	2.25	1.26	1.83	0.98	*F*(1, 291) = 8.03	.005
Allows boys to show that they are real men	2.22	1.32	1.91	1.06	*F*(1, 291) = 3.36	ns
Is a good way to discipline a woman	2.52	1.46	2.36	1.36	*F*(1, 291) < 1	ns
This is a man’s right when he has a girlfriend	2.30	1.26	2.12	1.17	*F*(1, 291) < 1	ns
Is justified because girls do not listen well to a boy’s need to have sex	2.60	1.36	2.19	1.18	*F*(1, 291) = 5.34	.022
This is not a problem if a girl visits a boy at his home	2.69	1.42	2.16	1.11	*F*(1, 291) = 9.95	.002
This is not a problem if a girl is dressed sexy	2.68	1.41	2.41	1.29	*F*(1, 291) = 1.54	ns
This is not a problem if the girl is financially dependent on the boy	2.29	1.26	1.99	1.08	*F*(1, 291) = 3.98	.047
For a boy to force a girl to have sex is wrong	3.23	1.51	3.50	1.46	*F*(1, 291) = 1.39	ns
If a boy forces a girl to have sex, he is a rapist	3.69	1.41	3.87	1.34	*F*(1, 291) < 1	ns
The boy may get arrested as it is against the law	3.82	1.33	3.91	1.28	*F*(1, 291) < 1	ns
The boy may have to go to jail	3.70	1.42	3.90	1.31	*F*(1, 291) = 1.02	ns

Absolute range for each variable, 1–5

**Table 3:** Gender differences in knowledge, social influence, and self-efficacy beliefs concerning forced sex

	Boys		Girls		*F*	*p*
	*M*	SD	*M*	SD		
Knowledge						
Forced sex is rape and is illegal in South Africa^a^	0.64	0.48	0.79	0.41	*F*(1, 668) = 15.49	<.001
There are special procedures to follow if you report that you have been raped^a^	0.66	0.47	0.79	0.41	*F*(1, 668) = 14.21	<.001
If you are raped, you should report what happened to the police who must open a case^a^	0.82	0.39	0.92	0.28	*F*(1, 668) = 12.58	<.001
If you are forced to have sex, you may be at risk of HIV^a^	0.79	0.41	0.91	0.29	*F*(1, 668) = 18.02	<.001
Social influence						
Among my friends, boys force girls to have sex to teach them a lesson^b^	2.24	1.09	2.24	1.04	*F*(1, 664) < 1	ns
Among my peers, boys force girls to have sex so that they do what they’re told^b^	2.32	1.10	2.23	1.01	*F*(1, 664) < 1	ns
In my family, men sometimes force women to have sex to show who is boss^b^	2.24	1.08	2.16	1.01	*F*(1, 664) < 1	ns
In my family, men force women to have sex if the women do wrong^b^	2.20	1.07	2.17	1.01	*F*(1, 664) < 1	ns
My friends will support a boy who forces a girl to have sex to make her listen to him^b^	2.23	1.04	2.09	0.99	*F*(1, 664) = 3.38	ns
My peers will support a boy who forces a girl to have sex to make her listen to him^b^	2.20	1.06	2.08	0.95	*F*(1, 664) = 1.88	ns
My mother will support a boy who forces a girl to have sex to make her listen to him^b^	2.01	0.98	1.91	0.93	*F*(1, 664) = 1.28	ns
My father will support a boy who forces a girl to have sex to make her listen to him^b^	2.07	1.05	1.88	0.95	*F*(1, 664) = 4.80	.029
Other family members will support a boy who forces sex to make a girl listen to him^b^	1.98	0.95	1.89	0.93	*F*(1, 664) = 1.09	ns
My teachers will support a boy who forces a girl to have sex to make her listen to him^b^	2.07	1.05	1.91	0.94	*F*(1, 664) = 3.31	ns
Friends would not respect a boy if he doesn’t force his girlfriend to have sex if she does wrong^b^	2.43	1.21	2.15	1.13	*F*(1, 664) = 7.81	.005
Peers would not respect a boy if he doesn’t force his girlfriend to have sex if she does something wrong^b^	2.41	1.23	2.21	1.13	*F*(1, 664) = 4.19	.041
Boys encourage one another to force their girlfriends to have sex^b^	2.44	1.20	2.54	1.17	*F*(1, 664) = 1.13	ns
To be “one of the boys” boys have to be able to say that they have forced their girlfriends to have sex^b^	2.45	1.13	2.47	1.06	*F*(1, 664) < 1	ns
Self-efficacy						
I feel confident that I will not force someone into sex when…						
I want to show who is boss^b^	3.24	1.47	3.17	1.40	*F*(1, 662) < 1	ns
I am drunk^b^	3.06	1.41	3.20	1.39	*F*(1, 662) = 1.22	ns
I like the person^b^	3.28	1.42	3.24	1.38	*F*(1, 662) < 1	ns
I am alone with my partner^b^	3.29	1.39	3.25	1.35	*F*(1, 662) < 1	ns
I am at my own house^b^	3.31	1.40	3.29	1.33	*F*(1, 662) < 1	ns
I am at a party^b^	3.23	1.41	3.30	1.35	*F*(1, 662) < 1	ns

^a^Dichtomized as 0 = not correct; 1 = correct

^b^Absolute range, 1–5

**Table 4:** Gender differences in intentions, cues to action, and action plans concerning forced sex

	Boys		Girls		*F*	*p*
	*M*	SD	*M*	SD		
Intentions						
I intend not to force my partner to have sex	3.45	1.36	3.62	1.19	*F*(1, 639) = 2.18	ns
I intend to have consensual sex	3.73	1.22	3.58	1.24	*F*(1, 639) = 2.60	ns
I intend not to force my partner to have sex when I am drunk	3.74	1.19	3.69	1.19	*F*(1, 639) < 1	ns
I intend not to force my partner to have sex when we are alone	3.71	1.21	3.70	1.14	*F*(1, 639) < 1	ns
I intent not to force my partner to have sex to show that I am a man	3.77	1.16	3.64	1.16	*F*(1, 639) = 2.36	ns
I intend not to force my partner to have sex at my own house	3.74	1.20	3.69	1.15	*F*(1, 639) < 1	ns
Cues to action						
The 16 days of activism targeting violence against women encourages me not to force girls to have sex	3.69	1.24	3.83	1.07	*F*(1, 639) = 1.75	ns
Action plans						
In the next 6 months, I plan to learn other ways of resolving conflicts, not by forced sex	3.79	1.17	3.95	1.06	*F*(1, 639) = 2.54	ns
In the next 6 months, I’m going to solve relationship problems by non-violent means, not by forced sex	3.79	1.16	3.99	0.98	*F*(1, 639) = 5.51	.019

Absolute range, 1–5

### Data Analysis

All analyses were done using SPSS 19.0. Descriptive analyses were undertaken to describe the sample. Attrition analysis was done using logistic regression with drop-out after 8 months as the dependent variable and demographics, sexual behaviors, knowledge, attitudinal beliefs, social influence, self-efficacy, and intention as independent variables. Concerning the gender differences, multivariate analyses of variance (MANOVA) were conducted per sociocognitive construct, while adjusting for sexual and forced sex experience by including these two variables as covariates. This was done to avoid answers that were biased because of the level of sexual experience and being forced into sex. We did not correct for the multilevel structure of individuals clustered within schools, since the overall intraclass correlation was low (.03) and school differences in forced sex prevalence were not significant. For the assessment of pairwise comparisons, we applied the Bonferroni method to correct for potential Type 1 errors due to multiple testing. We could not use the complete sample at follow-up (*N* = 582) for the longitudinal analyses, since half of the sample took part in an intervention to reduce gender violence. Therefore, any longitudinal results were obtained from the control group only (*N* = 386).

## Results

### Gender Differences in Demographic Characteristics and Sexual Behavior

Results showed that the boys were slightly older and had gone to bed hungry more often in the previous week (see Table 1). Boys started with sex at an earlier age than girls (14 vs. 15 years of age) and more boys were found to be sexually active (26 vs. 12 %). A greater proportion of boys reported to have been forced to have sex (4.9 %) as compared to the girls (3.3 %), but this difference was not significant (see Table 1). After 8 months, similar differences between boys and girls were present with 10.5 % (*N* = 12) of the boys and 6.4 % (*N* = 10) of the girls reporting to have been forced to have sex. This gender difference was not significant, *χ* ^2^(1, 270) = 1.49, but the reported increase in forced sex (the rates effectively doubled in 8 months) was significant for boys (McNemar’s test: *p* = .03), and borderline significant for girls (McNemar’s test: *p* = .06).

Concerning sexual behavior, we also found that, in general, students who had sexual experience were older, *t*(726) = 13.63, *p* < .001, and had higher chances of food insecurity (going to bed hungry in the previous week: *χ* ^2^(2, 635) = 8.47, *p* = .01. In addition, older students were more often found to be victims of forced sex than younger students, *t*(719) = 2.92, *p* < .01. No other associations between demographic factors and sexual behavior were found.

### Gender Differences in Beliefs Concerning Forced Sex

We assessed gender differences in beliefs concerning forced sex, while adjusting for individual differences in sexual experience and forced sex by including them as covariates. Using Pillai’s trace, there was a borderline significant difference between boys and girls in attitudinal beliefs, *V* = 0.07; *F*(13, 279) = 1.73; *p* = .055. Post hoc pairwise comparisons showed that boys associated forced sex more than girls with showing love for their partner and as a way to satisfy their sexual urges. Additionally, boys indicated stronger agreement that forced sex was justified when girls did not listen to a boy’s need for sex, when a girl visited a boy at his home, and when the girl was financially dependent on the boy (see Table 2).

When comparing the knowledge of boys and girls on the subject of legal procedures related to forced sex, Pillai’s trace showed that there was a significant gender difference, *V* = 0.04; *F*(4, 665) = 6.75; *p* < .001. The post hoc pairwise comparisons are summarized in Table 3 and showed that girls were significantly more knowledgeable than boys on all four questions: 79 % of the girls answered correctly that forced sex was identical to rape and therefore illegal, compared to only 64 % of the boys. Likewise, 79 % of the girls were aware that special procedures are to be followed when one has been raped, compared to only 66 % of the boys. There was slightly more consensus on the necessity of reporting rape to the police: 92 % of the girls versus 82 % of the boys agreed that rape should always be reported. A negative impact on one’s health, such as contracting HIV, was acknowledged by 91 % of the girls and 79 % of the boys.

Concerning social influences, the results showed that boys reported stronger social influences that approve forced sex than girls, *V* = 0.04; *F*(14, 651) = 2.15; *p* = .008. Pairwise comparisons showed that boys believed more strongly that their father would support a boy that uses forced sex to make a girl obedient and that friends and peers would not respect a boy if he didn’t force his girlfriend to have sex if she did something wrong (see Table 3).

Boys and girls also differed in their overall self-efficacy to not engage in forced sex, *V* = 0.02; *F*(6, 657) = 2.16; *p* = .045. However, post hoc pairwise comparisons did not show any difference on single items (see Table 3). Finally, boys and girls differed in their future intentions and action plans, *V* = 0.04; *F*(9, 631) = 2.98; *p* = .002. Pairwise comparisons showed that this was mainly due to the fact that girls were more likely than boys to indicate that they wanted to solve future problems in their relationships by non-violent means (see Table 4).

## Discussion

Physical and sexual violence are strongly associated with HIV/AIDS and unwanted pregnancy (Campbell, 2008; Dunkle et al., 2004; Jewkes et al., 2006b; Maman, 2002; Van Der Straten et al., 1998). The current study sought to explore self-reported forced sex in boys and girls in rural KwaZulu-Natal as well as their beliefs about forced sex. The results can be summarized in the following main conclusions.

First, the data indicated our age group is at risk of being forced to have sex, and that percentages doubled in the 8 months between baseline and follow-up. In total, 10.5 % of the boys had become a victim of forced sex and 6.4 % of the girls. Higher prevalence rates for young males concerning forced sex can also be found in the South African Youth Risk Behaviour Survey (Reddy et al., 2003). Mathews et al. (2009) reported on Grade 8 Cape Town high school students and found that at baseline 9 % of the girls and 24 % of the boys reported being the victim of physical violence in intimate relationships. Furthermore, 28 % of the boys and 18 % of the girls who had made the transition to first intercourse reported that they had been forced to have sexual intercourse. From those boys and girls who reported being victims of intimate partner violence at baseline, 36 % reported ever being forced to have intercourse at a second follow-up (13 months later), compared to 19 % of those who did not report physical violence at baseline. The 2008 Medical Research Council report indicated that among Grade 8–11 learners in KwaZulu-Natal (with age groups ranging from 13 to 19 years), 11 % of the boys and 7 % of the girls reported being forced to have sex (Reddy et al., 2010). The overall South African rates were 12 and 8 % for boys and girls, respectively. The report furthermore shows that, by the age of 19, 14 % of the boys and 11 % of the girls reported being forced into sex.

Little in-depth data are available explaining the higher prevalence rates of reported force sex among boys, of which there may be several explanations. First, in our study, boys reported much more sexual activity both at baseline (25.9 %) and at follow-up (29.8 %) than girls (12.0 % at baseline and 10.9 % at follow-up). Hence, involvement in sexual activities may also result in more exposure to situations where sex can be forced. Second, the students’ interpretation of forced sex may not only include being penetrated by someone else, but also being forced to have sex with others by peers (i.e., social pressure) or being seduced by girls. The fact that sexual relationships play an important role in the transition to adulthood increases the pressure felt by young boys to engage in sex. Third, it may be that female victims of forced sex drop out of school more regularly than boys do. The reason for this is that they have an increased risk of getting pregnant through their inability to negotiate about condoms or contraceptives (Freudenberg & Ruglis, 2007; WHO, 2002; Wood et al., 1998). Fourth, a complicating factor in the interpretation of these figures is the definition of forced sex. In some studies, this includes anal and vaginal sex whereas it may also include oral and thigh sex in other studies. Thigh sex, also referred to as intercrural sex, is a type of intercourse that is practiced in the Zulu culture. Courting couples could engage relatively freely in the practice of non-penetrative, thigh sex (*ukusoma* or *ukuhlobonga*). Men were fined for breaking an unmarried woman’s virginity and for causing pregnancy, but non-penetrative forms of sex were tolerated and even encouraged (Hunter, 2004). Clearly, more in-depth analysis concerning this phenomenon is needed.

Boys also reported a more positive attitude towards forced sex and more supportive norms from others that supported forced sex. The fact that boys, and even girls, did not score in the lower quartile concerning the belief that forced sex was a sign of love and that forced sex was justified if it happens in the boy’s home or as a way to discipline a woman was disquieting. This pattern was also visible in the relatively high scores of agreement on the acceptance of forced sex by peers and friends. It may be that the more positive beliefs of boys about forced sex stemmed from the social norms in South Africa and KwaZulu-Natal about masculinity and acceptance of sexual violence (Anderson, Ho-Foster, & Matthis, 2004; Buthelezi, 2006; Flisher et al., 2007; Jewkes & Morrell, 2010; Sathiparsad, 2008). Yet, despite the gender differences, our study also highlighted that, overall, girls also seemed to hold similar ideas about male sexual dominance. Concerning more proximal factors, both groups indicated equally self-efficacy in their perceptions about refraining from forced sex and concerning their intentions regarding using forced sex in various situations. However, boys were more likely to use forced sex as a means to solve relational problems. Overall, our data clearly suggest that beliefs about forced sex in both boys and girls need to be addressed in prevention programs.

Our study was subject to limitations. First, self-report about sensitive issues, such as forced sex, may lead to underreporting. Although anonymity was ensured, the fact that classrooms were often occupied with more than 40 students may have led to feelings of uncertainty and may have led to underreporting of forced sex problems. Second, definitions of forced sex differ from study to study. Although our study explicitly assessed whether the adolescent was ever forced to have sex and was forced to have sex in the past 6 months, and interpretations of the Zulu translation were checked to verify whether this referred to penetrative sex, we cannot fully rule out that different interpretations were made by the adolescents. This and the fact that more boys reported being forced into sex suggests a need for further in-depth (qualitative) research.

Will it be possible to reduce gender violence? Previous studies conducted in KwaZulu-Natal clearly indicate the need for such programs, as many male adolescents assert masculinity, largely through sexual intercourse with females and this is prioritized over health and safety (Sathiparsad, Taylor, & De Vries, 2010). Wood et al. (1998) suggest that gender violence has been particularly neglected in adolescent sexuality arenas and that future prevention programs should not only address the perceptions of boys concerning forced sex, but also address the perceptions of the (male) community that surrounds these young male adolescents. The South African Stepping Stones program successfully altered beliefs about gender and HIV risk, particularly among men, by offering viable alternative normative behaviors (Hayes, 2008). Stepping Stones also significantly improved a number of reported risk behaviors in men, with a lower proportion of men reporting perpetration of intimate partner violence across two years of follow-up and less transactional sex and problem drinking at 12 months (Jewkes et al., 2008). Furthermore, two South-African community-based HIV prevention programmes demonstrated reductions in intimate partner violence (Jewkes, Nduna, Levin, Jama, & Dunkle, 2007; Jewkes et al., 2006b; Pronyk et al., 2006). In order to reduce sexual violence, community participation is required to reduce the high rates of sexual violence which contribute to HIV transmission (Abrahams & Jewkes, 2005; Taylor, Meyer-Weitz, Jinabhai, & Sathiparsad, 2009). Mathews (2008) suggested a reciprocal determinism between individual-level factors and environmental factors and expressed the need for a social environment that was supportive of the behavior. Peer opinion leaders might be particularly suited to influence the attitudes and subjective norms correlated with partner violence (Flisher et al., 2007). Shifting male teacher’s attitudes towards gender violence is also crucial for creating conditions necessary for the achievement of gender equality (Bhana, 2009). Furthermore, a host of economic vulnerabilities may also underlie young women’s inability to challenge all sorts of sexual invitations and levels of forced sex. In the context of poverty, young women speak of money as the driving force for sex and relationships (Jewkes et al., 2008; MacPhail & Campbell, 2001; Pettifor et al., 2004; Swart-Kruger & Richter, 1997). Hence, poverty reduction is also crucial in the battle for empowerment for girls in South-Africa. Poverty reduction is also necessary to empower men so that sexual conquest is not the only way to demonstrate their masculinity (Hunter, 2004). Finally, although not the focus of our study, other studies indicate that alcohol independently influences decisions around sex and undermines skills for condom negotiation and correct use. Thus, not surprisingly, people with problem drinking in Africa have a twofold higher risk for HIV than non-drinkers. Also, sexual violence incidents often coincide with heavy alcohol use, both among perpetrators and victims. Reducing the harm of alcohol necessitates both population- and individual-level interventions, especially raised taxation, regulation of alcohol advertising and provision of “Brief Interventions” by all healthcare workers (Chersich & Rees, 2010).

In conclusion, our data showed that gender violence and related beliefs seem to be quite accepted by our male and female study population. The data suggest that several beliefs need to be addressed in prevention programs in order to reduce gender violence. This approach should not only address the youngsters themselves but also the larger community (Jewkes & Morrell, 2010), and should be sensitive to poverty problems.
